# Coarse-Grained Monte Carlo Simulations with Octree Cells for Geopolymer Nucleation at Different pH Values

**DOI:** 10.3390/ma17010095

**Published:** 2023-12-24

**Authors:** Nicolas Castrillon Valencia, Mohammadreza Izadifar, Neven Ukrainczyk, Eduardus Koenders

**Affiliations:** Institute of Construction and Building Materials, Technical University of Darmstadt, Franziska-Braun-Str. 3, 64287 Darmstadt, Germany; castrillon@wib.tu-darmstadt.de (N.C.V.); koenders@wib.tu-darmstadt.de (E.K.)

**Keywords:** 3D off-lattice coarse-grained Monte Carlo, aluminosilicates, metakaolinite-based geopolymer, alkali silicate solution, nucleation, cluster size distribution, pore-size distribution, pH

## Abstract

Geopolymers offer a potential alternative to ordinary Portland cement owing to their performance in mechanical and thermal properties, as well as environmental benefits stemming from a reduced carbon footprint. This paper endeavors to build upon prior atomistic computational work delving deeper into the intricate relationship between pH levels and the resulting material’s properties, including pore size distribution, geopolymer nucleate cluster dimensions, total system energy, and monomer poly-condensation behavior. Coarse-grained Monte Carlo (CGMC) simulation inputs include tetrahedral geometry and binding energy parameters derived from DFT simulations for aluminate and silicate monomers. Elevated pH values may can alter reactivity and phase stability, or, in the structural concrete application, may passivate the embedded steel reinforcement. Thus, we examine the effects of pH values set at 11, 12, and 13 (based on silicate speciation chemistry), investigating their respective contributions to the nucleation of geopolymers. To simulate a larger system to obtain representative results, we propose the numerical implementation of an Octree cell. Finally, we further digitize the resulting expanded structure to ascertain pore size distribution, facilitating a comparative analysis. The novelty of this study is underscored by its expansion in both system size, more accurate monomer representation, and pH range when compared to previous CGMC simulation approaches. The results unveil a discernible correlation between the number of clusters and pores under specific pH levels. This links geopolymerization mechanisms under varying pH conditions to the resulting chemical properties and final structural state.

## 1. Introduction

The “geopolymer” or alkaline aluminate silicate geopolymer material coined by Davidovits in 1978 [1] refers to materials synthesized through the combination of aluminosilicate powder mixed with potassium silicate or sodium silicate solution precursor [2,3,4]. Silicate solution precursors play a crucial role in initiating the alkalinization process. In this dissolution-precipitation geopolymerization reaction, the silicate [5] and aluminum bonds within the aluminosilicate powder solid material are first broken by hydroxyl (OH^−^) groups present in the alkaline solution. This is followed by a polycondensation reaction, ultimately leading to the formation of an aluminosilicate network [6]. The network consists of interconnected aluminum (Al) and silicate (Si) tetrahedra linked by oxygen-bridging bonds [3]. The utilization of gels or zeolites in the cement industry is becoming increasingly prevalent due to their inorganic structure and mechanical properties [7]. The kinetics of aluminosilicate-fluid interactions carry significant implications for various environmental and engineering processes, including CO_2_ sequestration, soil evolution, pollutant disposal, catalysis, adsorption, and petroleum drilling operations [8]. In addition, geopolymers are inherently fire resistant and have demonstrated excellent thermal stability, far superior to that of traditional cements [9]. These attributes render them highly attractive for numerous applications. Alkali aluminate silicate geopolymer materials demonstrate mechanical properties like typical Portland cement but require approximately 80–90% less CO_2_ during production [10]. Furthermore, they demonstrate exceptional resistance to acids compared to Portland cement, making it one of the most notable advantages associated with geopolymers [11,12,13,14]. However, questions regarding their durability and structural development remain open and can only be addressed through advancements in experimental resources and simulation techniques [10].

Most recently, Izadifar et al. [15] used the 3D off-lattice coarse-grained Monte Carlo (CGMC) approach to simulate the polymerization of alkaline aluminosilicate gels, their nanostructure particle size, and their pore size distribution. For this, the Gibbs free energy of dimerization reactions for the four different monomer species was used as the input table, taken from the literature computed through the DFT modeling method by White et al. [10,16]. In this way, the polymerization reaction for the silicate monomers presented as particles in the silicate-activated system was subjected to reach the equilibrium condition with an energy of 934 kJ/mol within 1,000,000 iterations. It has been reported that 91.80% of silicate particles presented in the cluster formation. Then, the metakaolinite [17] sub-system was involved in the MC particle selection and movement process to reach the equilibrium condition for seven million more iterations. At the equilibrium condition, 66.50% of particles participated in the cluster formation. In addition, Izadifar et al. [15] recently computed the enthalpy activation energy (Δ*H*^∗^) at far-from-equilibrium conditions, which is based on the transition-state theory (TST) for the calculation of atomistic reaction rates for silicate tetrahedra dissolution in MK through the DFT computational approach [18,19,20,21]. Most published studies on geopolymer systems have focused on fly ash/blast furnace slag composite systems, and, in most cases, the examination has been limited to observing X-ray diffractograms and ultimate compressive strength, which are standard techniques in cement science. Even though the information was collected using this experimental technique, it is still difficult to fully report for the alkali activation process quantitatively, primarily due to the multiple chemical mechanisms occurring [22]. The complexity of this field stems from the multiple simultaneous processes occurring in alkaline silicate systems and the existing limitations in applying comprehensive multiscale models. Previous theoretical and experimental investigations of aqueous silica chemistry under alkaline conditions have helped to elucidate the individual processes fundamental to the holistic behavior of silica in zeolite formation. However, conventional atomistic theoretical models fail to reproduce the mechanisms at the mesoscale level, mainly due to the large computational demands inherent in atomistic modeling over the required range of length scales [23]. Indeed, the precise intricacies encompassing the transformation process from an aluminosilicate precursor in alkali environments to a geopolymeric gel remain unverified both experimentally and theoretically, owing to the inherently intricate character of this mechanism. Consequently, a notable gap persists within the scholarly discourse concerning the intricate mechanistic depiction of the geopolymerization reaction [10]. The essential chemical and structural properties of geopolymers derived from metakaolinite, fly ash, and slag are investigated in terms of the impact of raw material selection on the properties of the geopolymer composites [3]. The use of geopolymer is a potential alternative to ordinary Portland cement, although the chemical and mechanical constitution of alkali activated mixes (AAMs) have not been understood and the pore structures of these materials have remained unexplored [10,18,19]. As a result, there is a great research opportunity to further investigate this topic. The crucial process of geopolymerization, which has only been briefly investigated thus far, involves the transformation of liquid precursors into a “solid” gel and densification structure. This transformation is key to controlling the nanostructure and porosity of geopolymers, enabling them to meet specific field requirements [21,23,24].

The main objective of this work is to implement a 3D off-lattice CGMC simulation of a coarse-grained model for studying the nucleation of alkaline aluminosilicate gel for three different silicate-activated systems. Based on the Gibbs free energy of dimerization reported by White et al. [10] for four different monomer species of Si(OH)_4_, Al(OH)_4_^−^.Na^+^, SiO(OH)_3_^−^.Na^+^.3H_2_O, and SiO_2_(OH)_2_^2−^.2Na^+^.6H_2_O, and considering the limitation of the tetrahedral structure for the particle polymerization in the system, a 3D off-lattice CGMG approach is adopted. Each monomer species is represented as a distinct coarse-grained particle type to compute the gel structure evolution as a function of the different number of iterations. In this manner, three different pH values (11, 12, and 13) have been investigated, incorporating a larger simulation system to obtain more accurate results using Octree cell expansion. The total energy of the system is calculated at various iterations, enabling a comprehensive understanding of its behavior. Additionally, the evolution of cluster formation and metakaolinite dissolution is studied over a total simulation period of 56 million iterations. The resulting structure is then analyzed to determine the distribution of cluster sizes and the characteristics of the pore network.

## 2. Simulation Model and Method

CGMC simulations were carried out using the canonical ensemble (NVT), which maintains a constant number of particles, volume, and temperature. The model utilized is a simple cubic off-lattice model, where the particles can freely move in all three dimensions (x, y, and z). It is important to note that much of the DFT/CGMC methodology has been thoroughly discussed and validated in previous research on silicate systems [15]. Therefore, to maintain brevity, we have not reproduced all that information here. However, there are significant differences between our current study and our previous work, which we outline below, along with a summary of the model implementation.

### 2.1. Specification of Silicate Particles in Each System

The solubility of silicate in solution is strongly affected by the pH. Previous research on solubility in aqueous solutions has shown that there is a Si-pH region where the solutions are homogeneous, but there are also other regions where the solutions exhibit more energetic reaction equilibria (please refer to Figure 1 in [25]). The condensation (polymerization) reaction was delineated through particle bonding, aiming to minimize the overall system energy. To facilitate this objective, the input table harnessed the Gibbs free energy pertaining to dimerization reactions across the four distinct monomer species, as derived from the literature [10] and obtained via the density functional theory (DFT) modeling method. The simulation can be summarized in two main parts: In the first part, silicate monomers, acting as activators of the system, are iterated until an equilibrium condition is reached. For the second part, the metakaolinite particle is enabled to participate in the dissolution process. For both parts, it is necessary to determine the number of silicate monomers that are present throughout the entire simulation for the systems with pH values of 11, 12, and 13. The percentage distribution of these silicate monomers is determined based on the silica diagram as a function of pH (refer to Figure 5 in [25]). In alkaline aqueous solutions, the equilibrium distribution of silicate species is significantly influenced by deprotonation equilibria, with monomer deprotonation constants defined by the following reaction equations and equilibrium constants [25]:

$${SiO}_{i-1}{\left(OH\right)}_{5-i}^{\left(i-1\right)-}\overset{K_{m}^{i}}{↔}{SiO}_{i}{\left(OH\right)}_{4-i}^{i-}+H^{+}$$

$${pK}_{m}^{1}=9.84-1.022\frac{I^{\frac{1}{2}}}{1+I^{\frac{1}{2}}}+0.11I$$

$${pK}_{m}^{2}=13.43-2.044\frac{I^{\frac{1}{2}}}{1+I^{\frac{1}{2}}}+0.20I$$

where ionic strength (*I*) is expressed in mol/L.

During the simulation, this percentage of monomer particles is controlled every 30 iterations to ensure the same protonation state throughout the process. The percentage of water, as well as Na cation, silicate in solution, and metakaolinite, are extracted from the data published by White [10] based on the selected silicate-activate system, and the exact values are listed in Table 1. To ensure the same volume in the three different systems (pH values are equivalent to 11, 12, and 13), the volume is calculated from the total number of particles, taking pH 11 as the reference. This ensures that the systems are the same size, even though the concentration and diameter of the particles are different, giving an advantage in comparing the pore size distribution between the three systems. Given the presence of three different silicate particles in each system, characterized by variable sizes and whose quantities depend on the pH level, the particle count within each system naturally diverges. To maintain a uniform solid volume in the three systems, each system consistently uses an identical percentage of silicate activator initialized at the beginning of the simulation, while the proportion of metakaolinite particles is systematically adjusted. This ensures that the systems have the same size, even if their concentration and particle size are different. Table 2 shows the total number of particles in the solution for the three different silicate monomers: Si(OH)_4_, SiO(OH)_3_^−^.Na^+^.3H_2_O, and SiO_2_(OH)_2_^2−^.2Na^+^.6H_2_O.

All systems are formed by a precursor (layered metakaolinite) surrounded by activating particles (three different types of silicate). Table 3 explains, in detail, the amount of each silicate particle for each system.

### 2.2. Monte Carlo Approach: Implementation in MATLAB Code

**Figure 1:**
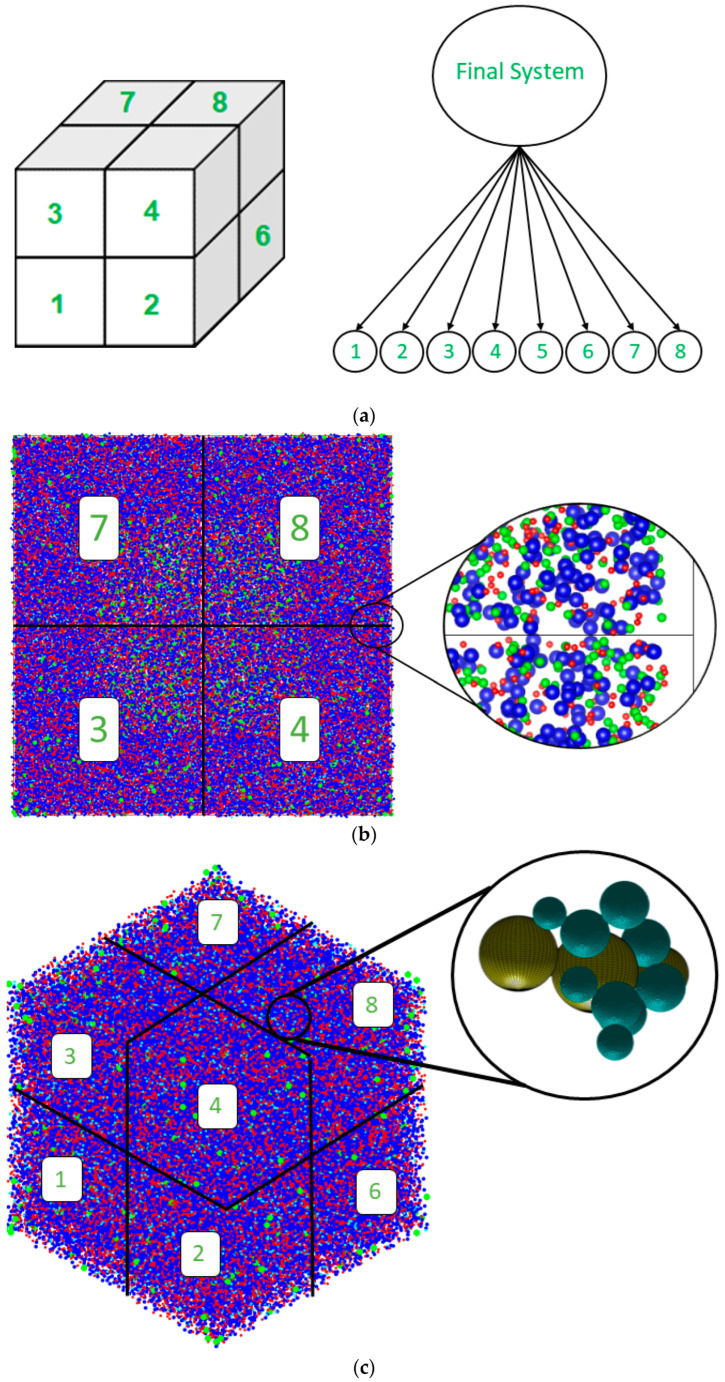
(**a**) The total system is divided into eight partitions, each representing a subsystem, following an Octree pattern. (**b**) Top view of the pH11 system, showing a detailed zoom-in of the interface planes within the octa-three simulation cell structure. Particles positioned at the periphery of each sub-system effectively percolate, connecting the adjacent cells, increasing the interconnectivity behavior of the total system. (**c**) The final status of the simulation at pH 11. The particles, clusters, and pore distribution are on the left side. Details are illustrated on the right side about the pore size distribution calculation: the dark aquamarine-colored particles represent the coarse-grained monomer particles (no distinction about the type), and the yellow particles represent the pore sizes.

An additional MATLAB code [26,27,28,29] was developed to facilitate the storage and analysis of data within individual cells, as illustrated in Figure 1a. To incorporate information from all eight neighboring cells, a binding definition of neighbor relationships was employed, considering two sites as neighbors only if they shared a common face; adjacency through an edge or a corner was assumed to be insufficient for classification. This approach was instrumental in ensuring comprehensive interactions among all cluster groups and monomers across cells. In Figure 1b, a detailed view is added where two subsystems are sharing particles that participate in the geopolymerization process. The software application provided detailed data throughout the simulation iterations, encompassing monomer specification, cluster dynamics, system energy fluctuations, and the complex metakaolinite dissolution process. Post-simulation analysis involved the utilization of the global scan method for extracting the cluster size distribution. A refined pore network model was subsequently constructed, assuming idealized spherical pores where monomers and dimers are considered aqueous species, i.e., part of the solution phase reported by White et al. [10]. The digitization of particle structures was accomplished through the implementation of a watershed algorithm and a city-block distance transform function (Figure 1c). Subsequently, pore connectivity and pore size distribution were deduced utilizing these techniques [15]. Consequently, the total system energy was computed based on the Gibbs free energy of dimerization, as reported by prior research [10]. This energy quantification is naturally linked to the number of particles within each system, which varies among the different pH environments. To ensure the precision and comparability of data between systems, an energy calculation correction factor was introduced. Specifically, the system with the highest particle count (pH 11) served as the reference point. This reference point (pH 11) was divided by the total number of particles for each system, and this ratio was subsequently applied to adjust the energy values computed during the simulation for each system.

### 2.3. Octree Cell Approach: Development of the MATLAB Program

Atomistic theoretical models are usually unable to reproduce the mechanisms taking place at the mesoscale level in zeolites due to the significant computational demands associated with atomistic modeling over the required length scales [2]. The primary objective is to investigate particle behavior during a polymerization process using a simple mechanical model. This investigation is crucial for understanding nanoparticle formation, as larger system sizes are essential to achieve statistically significant results. Additionally, simple models facilitate a more focused analysis of specific features of the system [30]. The simulation methodology employed in this study was based on our previous research [15], but with a notable improvement in system size, which was increased from 200 × 200 × 200 Å^3^ to 400 × 400 × 400 Å^3^. To accomplish this, an Octree cell with the first level of partition was developed, enabling parallel simulation processes, which are typically available in high-performance computing (HPC) environments. Specifically, eight identical programs with 7,000,000 iterations were executed simultaneously, and their results were integrated at the end of the simulation for comprehensive structural analysis (as explained in Section 2.2). The utilization of Octree patterns significantly reduced the memory requirements of the CGMC solver during the simulation of large-scale systems. This approach strikes a balance between minimizing artificial effects in small lattice simulations and achieving convergence within a reasonable timeframe.

### 2.4. Density Functional Theory (DFT) Calculation

**Figure 2:**
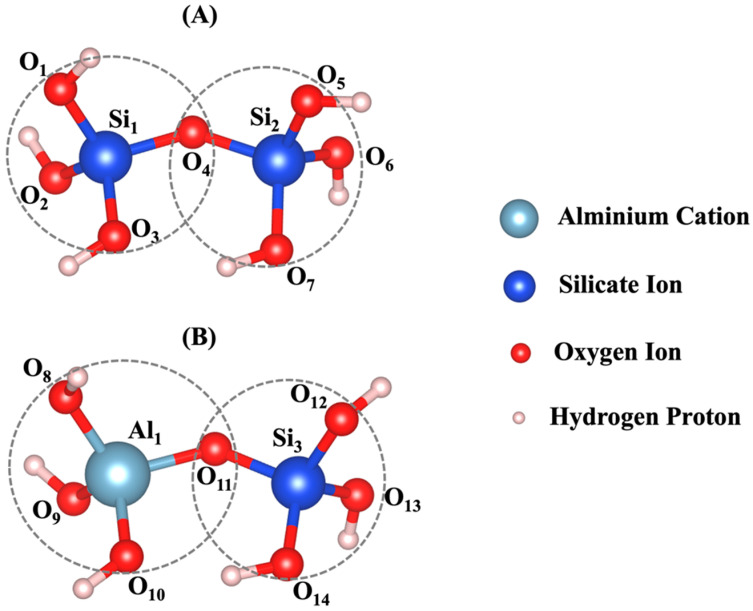
Dimerization reaction based on tetrahedron formation for (**A**) Si-O-Si and (**B**) Si-O-Al optimized by the DFT computational approach. Table A1 and Table A2 in Appendix A show bond lengths and angles between atoms. The radius of coarse-grained particles equals the averaged bond length. The two particles touch at a single point, either the center of bonding oxygen, O4 (case (**A**)) or O11 (case (**B**)).

To pass the geometrical parameters of the aluminate and silicate monomers (Figure 2), the density functional theory (DFT) calculations [31] were carried out. The Vienna ab initio simulation package (VASP) [32,33,34,35,36] employed the projected-augmented wave (PAW) method [37] and pseudopotential to define electron-ion interaction. The electron exchange and correlation functional were chosen in the generalized gradient approximation (GGA) with the Perdew−Burke−Ernzerhof (PBE) parametrization [38]. The Brillouin zone was sampled using a well-converged k-sampling equivalent given by 1 × 1 × 1 Monkhorst-Pack *k*-points for the total system [39]. A well-converged plane-wave cutoff energy of 400 eV was employed for the structural relaxations. The break condition of 10^−6^ eV was set for the convergence criterion for the electronic self-consistent cycles. In these calculations, the ions were relaxed until the forces were lower than 10^−3^ eV/Å. A three-dimensional visualization software (VESTA) was also utilized for the structural analysis of our models [40].

## 3. Results and Discussion

**Figure 3:**
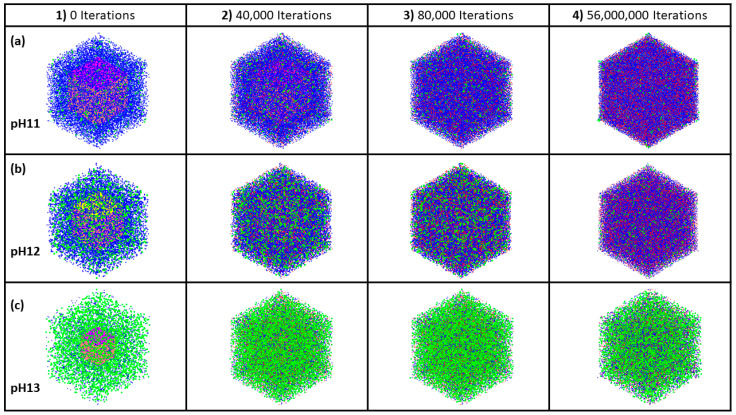
The evolution of the structures and cluster formation for the three different geopolymer systems extracted at a certain number of iterations of 0, 40,000, 80,000, and 56,000,000, respectively. The point at which the metakaolin is added to the system is denoted at iteration 0, with pre-equilibration taking place from iteration “8,000,000” to iteration 0. (**a**–**c**) (**a**) Represents the system with a pH of 11, while (**b**) illustrates the system at pH 12, and finally, (**c**) depicts the system at pH 13.

**Figure 4:**
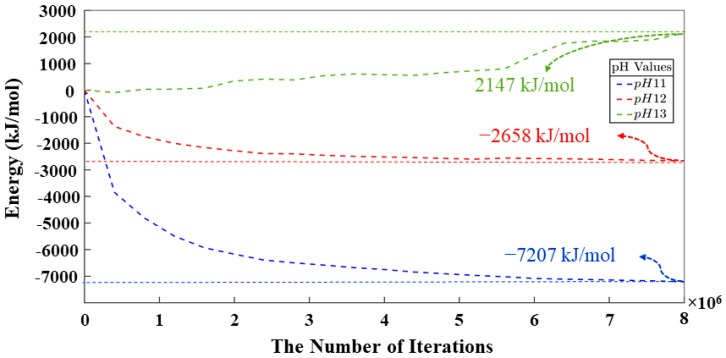
Energetic evolution of silicate particles in solution obtained for pH systems of 11, 12, and 13, where metakaolinite is not yet involved.

Table A1 and Table A2 in the Appendix A show bond length and angles between atoms, respectively. According to the DFT computational method, β angles of 135.12 and 138.28 degrees have been computed for the dimerization reaction for Si_3_-O_11_-Al_1_ and Si_1_-O_4_-Si_2_ (Figure 2) based on tetrahedron formation, respectively. The average bond lengths of 1.65 and 1.76 angstrom were considered for Si-O and Al-O in tetrahedra monomer formation, defining the coarse-grained particle radius for each particle type, respectively. The average angles of 107° and 108° were considered for O-Si-O and O-Al-O in tetrahedra monomer formation, defining each particle combination, respectively. Polycondensation unites the monomers, causing them to touch at a singular point—the center of bonding oxygen, shared by both particles (Figure 2). The images of the evolution of the three geopolymer systems during the entire process are shown in Figure 3. The different monomer building units (coarse-grained particles) are depicted with the following color codes: Si(OH)_4_ is given in cyan; SiO(OH)_3_**^−^**.Na^+^.3H_2_O in blue; SiO_2_(OH)_2_^2−^.2Na^+^.6H_2_O in green; and Al(OH)_4_.^−^Na^+^ in red. Figure 3a–c shows the process for systems with pH 11, 12, and 13, extracted at a certain number of iterations of 0, 40,000, 80,000, and 56,000,000, respectively. For systems 11 and 12, between iterations 40,000 and 80,000, there is a dissolution process of the metakaolinite particle, which is no longer clearly observed because it is in the middle of the system; this process continues until iterations 382,400 and 220,000 are reached, at which point the precursor particle is completely dissolved for the respective systems. For the system with pH 13, at iteration 45,600, the precursor is completely dissolved, and at iteration 80,000, the condensation process begins. At iteration 56,000,000, the final equilibrium condition is observed for pH systems 11, 12, and 13, with a percentage of particles involved in the formation of the gel of 66.46, 65.79, and 33.59%, respectively. Figure 4 illustrates the equilibrium conditions observed during the energy computation for the silicate-activated system in three distinct pH systems following 8 million iterations within the solution, with the exclusion of metakaolinite. As explained in our recent study [15], this process is performed to reach an equilibrium condition with dissolved activator silicates at the beginning of the simulation. Taking into consideration the variation in particle concentrations across the three systems, an equivalent energy was computed based on the number of particles (see Section 2.2). This calculation was undertaken to analyze fluctuations in energy and enable a meaningful comparison between the system’s behavior. Observations revealed that equilibrium conditions characterized by energy convergence are achieved after 8 million iterations for the systems with pHs 11 and 12. Conversely, for the system with pH 13, a more heterogeneous curve unfolds throughout the process, owing to the predominant molecule types encapsulated within this system. The discrepancies in energy profiles among these systems are attributed to the collective involvement of particles in the polymerization process, as determined by DFT. As depicted by the results for pH systems 11, 12, and 13, featuring respective values of −7207 kJ/mol, −2658 kJ/mol, and 2147 kJ/mol, the situation diverges notably in the pH 13 system due to the prevalent monomer types and their distinct Gibbs free energy characteristics. In this context, it is noteworthy that the formation of clusters occurs even between particles not inherently predisposed to perform polymerization. This phenomenon appears from the inclusion of a probability calculation in the computational code, which activates when the energy fails to decrease and instead exhibits an increase (refer to [15] for details).

**Figure 5:**
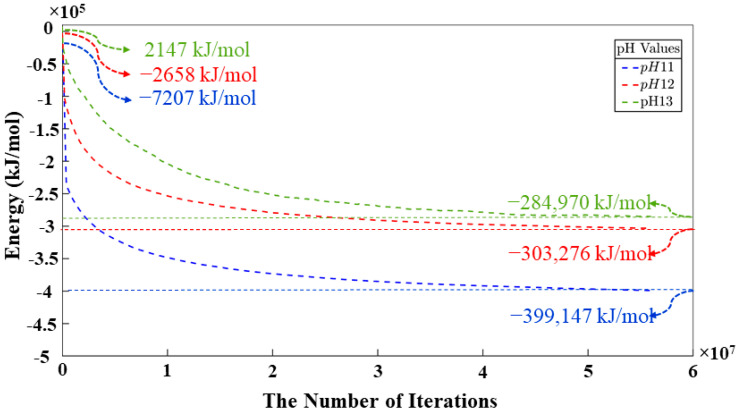
The equilibrium condition for three different pH systems was obtained through energy minimization computation of the silicate particles (starting at 0 iterations) and metakaolinite for 8 million iterations. The point at which metakaolinite is introduced to the system is denoted at iteration 0. Thus, pre-equilibration takes place for additional “8,000,000” iterations (shown in Figure 3).

According to Figure 5, the point at which the metakaolinite is “added” to the system is denoted at iteration 0, with pre-equilibration taking place from iteration “8,000,000” to iteration 0. Therefore, the energy at the beginning of the simulation is not zero, owing to the ore-equilibrium process involving the silicate monomers present in the solution as activators. This energy trend remains consistent across the three pH systems (11, 12, and 13) displayed in Figure 5. It signifies a rapid decline in energy, indicative of metakaolinite particle dissolution and the commencement of the polymerization process, during which aluminum monomers from the metakaolinite particle become integral to the process. After 40 million iterations, the energy trend starts to become more stable, and by 56,000,000 million iterations, the energies for pH systems (11, 12, and 13) converge to values of 399,147 kJ/mol, 303,276 kJ/mol, and 284,970 kJ/mol, respectively. Given that these three systems share equivalent dimensions but differ in particle quantities, an energy correction was applied to ensure a precise correlation among their values.

**Figure 6:**
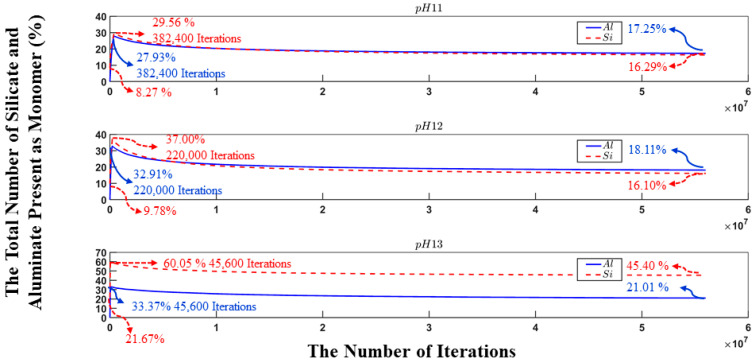
The change in the number of aluminate and silicate monomers present in the system during 56 million iterations for the different pHs. Metakaolinite particles are considered monomers only when dissolved according to the dissolution process.

Figure 6 is plotted to illustrate the evolution of the percentage of silicate and aluminate monomers in the three distinct pH systems over the course of the simulation. At the beginning of the process, the presence of aluminate monomers remains zero, as all aluminate particles are initially confined within the metakaolinite particle. Conversely, the total silicate monomers have an initial percentage at the beginning of the simulation: 8.27, 9.78, and 21.67% in the systems with pHs 11, 12, and 13, respectively. These initial values are indicative of the silicate monomer content within the solution. The above-mentioned results provide insights into the polymerization dynamics, particularly highlighting the less pronounced polymerization observed in the pH 13 system as a result of the sum of binding energies between the different particles, which are predominantly found in the system, being less prone to the condensation process. As the simulation advances, there is a rapid increase in the quantities of silicate and aluminate monomers during the precursor dissolution. The iterations at which the highest amount of aluminate (Al(OH)_4_^−^.Na^+^) and silicate monomers (Si(OH)_4_, SiO(OH)_3_^−^.Na^+^.3H_2_O, and SiO_2_(OH)_2_^2−^.2Na^+^.6H_2_O) are observed in each system coincide with the iterations marking the complete dissolution of the metakaolinite particle. These values were obtained after 382,400, 220,000, and 45,600 iterations concerning the pH values of 11, 12, and 13, respectively. However, at iterations of 11,333,600 (pH 11) and 5,802,400 (pH 12), the proportion of aluminate monomers exceeds that of silicate monomers, and this relative ratio remains the same until the end of the simulation. In contrast to these systems, in the system with pH 13, the amount of silicate monomers remains superior compared to that of aluminate monomers throughout the simulation, without any significant changes.

**Figure 7:**
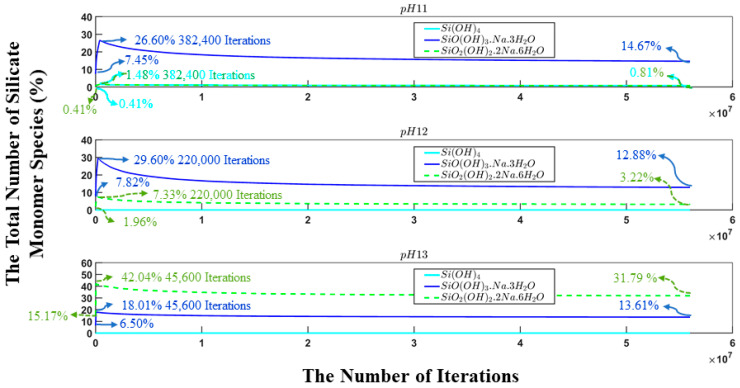
The evolution of the number of silicate monomers present in the system during 56 million iterations. At the start of the simulation (at iteration zero), metakaolinite particles were not included as they were undergoing the dissolution process.

For a better understanding of the behavior of silicate monomers over the number of iterations, Figure 7 has been plotted to specify the amounts of each silicate monomer present as a single particle in three different pH systems (11, 12, and 13) during the simulation process. This chart enables precise control over the program’s outcomes, offering a clearer understanding of the specific instant during the process when these particles reach their maximum concentration. This marks the conclusion of the dissolution phase and the commencement of the polymerization process. The three different types of monomer species, namely Si(OH)_4_, SiO(OH)_3_^−^.Na^+^.3H_2_O, and SiO_2_(OH)_2_^2−^.2Na^+^.6H_2_O, contributed 0.41, 7.45, and 0.41% (total 8.27%, as illustrated in Figure 5) at the commencement of the simulation for pH system 11, respectively. In the case of pH system 12, the same previously monomer species contributed 0, 7.82, and 1.96% (totaling 9.78%, as depicted in Figure 5), respectively. For the final system with a pH of 13, the contributions of the previously elucidated monomers are 0, 6.50, and 15,17% (total 21.67%, as illustrated in Figure 5), correspondingly.

**Figure 8:**
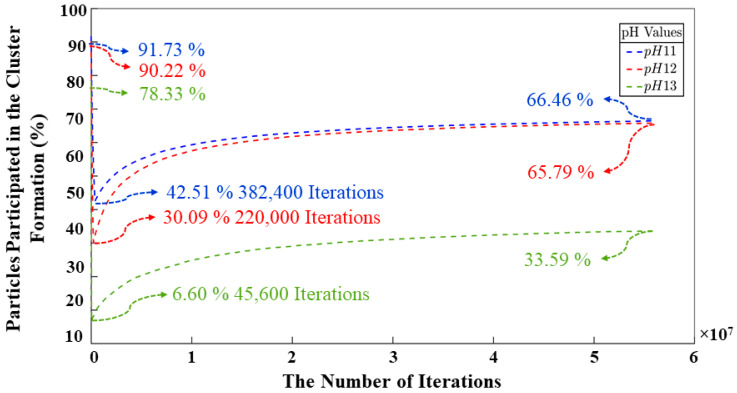
The evolution of cluster formation in the three systems during 56 million iterations.

Figure 8 was plotted for a better understanding of the dynamics governing the polymerization process across all systems during the 56 million iterations. This figure can be approximately divided into two distinct regions: the first region (before ~10^3^ iterations) is primarily dominated by the dissolution of the aluminate and silicate particles, although the second region (after ~10^3^ iterations) is dominated by the formation of the geopolymer gel. At the initial stage, the percentage of aluminate and silicate monomers involved in the condensation process for systems 11, 12, and 13 is 91.73, 90.22, and 78.33%, respectively (excluding metakaolinite monomers). It can also be noted from Figure 5 that the presence of the remaining monomers, which did not participate in the process, is about 8.27, 9.78, and 21.67% for the corresponding systems 11, 12, and 13. As the iteration process advances, this percentage of cluster evolution decreases, which means that the dissolution process of the metakaolinite particle has begun. The percentage of monomers involved in the gel formation reaches a minimum in the iterations 382,400, 220,000, and 45,600, with a percentage of 42.51, 30.09, and 6.60% for systems 11, 12, and 13, properly. At this point, the polymerization process begins; moreover, at the end of the polymerization and reorganization processes, the total percentage of particles that participated in the gel formation is 66.46, 65.79, and 33.59% for each respective system. It is interesting to note that the proportion of monomers involved in the evolution of cluster processes towards the culmination of the simulation within the pH 11 and pH 12 environments exhibits a near double increase when contrasted with the pH 13 system. Remarkably comparable outcomes are also perceptible during the concluding stages of the processes in the pH 11 and pH 12 systems. Thus, the pH affects, via the initial monomer speciation, the process of polycondensation and reorganization of those species within the geopolymer gel.

**Figure 9:**
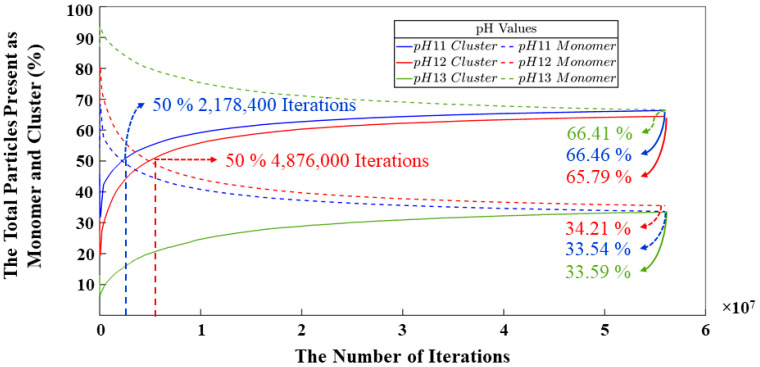
The change in the number of monomers participating and not participating in the cluster formation during 56 million iterations.

**Figure 10:**
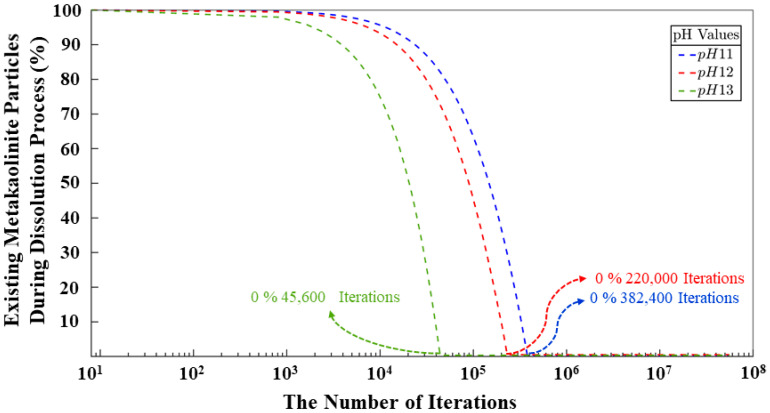
Dissolving process of metakaolinite during 56 million iterations.

Figure 9 shows a combination of monomers and clusters through the iterations. Once the simulation is started, the silicate and aluminate particles are continuously dissolved in the solution until the system is supersaturated. It should be noted that the dissolution region is relatively short compared to the iterations. In Figure 9, it is possible to analyze the point at which the monomers and the cluster reach the same percentage for the pH 11–12 systems. With values of 50% and 2,178,400 iterations for pH 11, and the same percentage but with more iterations (4,876,000) for the pH 12 system. For the pH 13 system, this point is never reached as particles are not so susceptible to carry out the condensation and reorganization processes. The model with the fewest number of iterations needed to reach the equilibrium state between particles that participated and those that did not participate in the polymerization process is the pH 11 system. This system has the highest number of particles but requires fewer iterations compared to the other models. The reason is the percentage of silicate monomers that must be maintained throughout the simulation and their energy values, which are more favorable for the geopolymer gel formation. While it holds true that the system requiring a smaller number of iterations to attain equilibrium between particles engaged in cluster formation and those not involved in said process is the pH 11 system, it is noteworthy to observe a strikingly analogous pattern in the evolution of the cluster dynamics between the pH 11 and pH 12 systems throughout the entirety of the simulation, with a difference of 0.67%, as depicted in the graph. At the culmination of the simulation, the results indicate values of 66.46, 65.79, and 33.59%, representing the percentage of particles engaged in gel formation for systems with pHs 11, 12, and 13, respectively. Conversely, three percentages of 33.54, 33.21, and 66.41% were attributed to particles that remain uninvolved in any polymerization processes. Figure 9 further displays a very high percentage of SiO(OH)_3_^−^.Na^+^.3H_2_O and SiO_2_(OH)_2_^2−^.2Na^+^.6H_2_O monomer contents for the pH 13 system after the first 8,000,000 iterations. This is due to the incompatibility of these two particles to perform the condensation process; this has already been discussed in other sections of this article. This results in a larger pore volume at the end of the process. Figure 10 shows the evolution of the metakaolinite particle throughout the process for the three different systems. The decrease in particle size is an indication of the degree of dissolution of the precursor particle, with both silicate and aluminate species being released back into the solution until the solution is supersaturated. The systems show the same trend, and at iterations 382,400, 220,000, and 45,600, the precursor is completely dissolved for pH systems of 11, 12, and 13, respectively.

**Figure 11:**
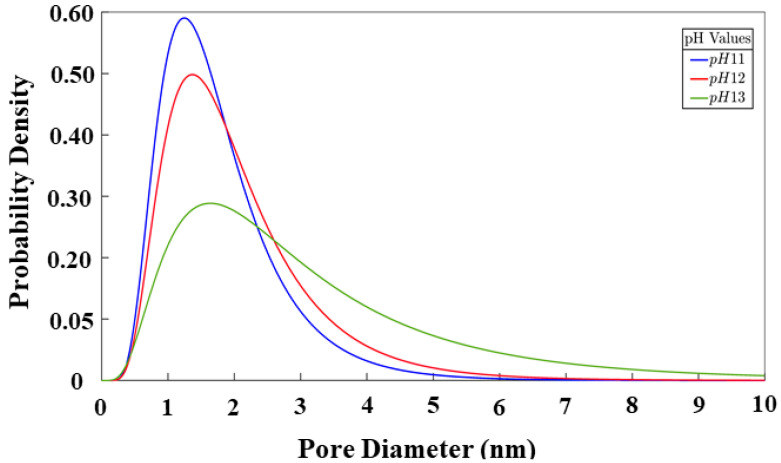
Cluster size distribution after 56 million iterations.

Figure 11 illustrates the distribution of the pore sizes for all the systems. In our recent study [10], the results of the pore analysis were compared with the results of Yang [11], showing the closeness of our results to the results obtained by them through the experimental methods. The focus on this model now is to be able to analyze this behavior at a microporous level between the three different systems to visualize the influence of the monomer type on the final state of the geopolymer gel. The pores in the three systems are comprised as follows: The pH 11 system features a 1.26 nm pore diameter region with a probability of 59.03%. Meanwhile, the pH 12 system displays a region with a 1.39 nm pore size, comprising 49.80% of the probability density. Finally, the pH 13 system, which is the one containing the least number of particles (see Table 2), has a higher probability of 28.84% for a pore size of 1.67 nm. The differences in the curves allow observing the influence that pH has on the final composition of the gel, making it clear that it is a value that directly affects its structure. For the system with pH values of 11, and 12, a tendency for coarsening its final pore structure is observed. For the system shown in the green-colored graph (pH 13), a tendency to contain pores with larger diameters is noted, attributed to its higher percentage of monomers at the end of the simulation.

**Figure 12:**
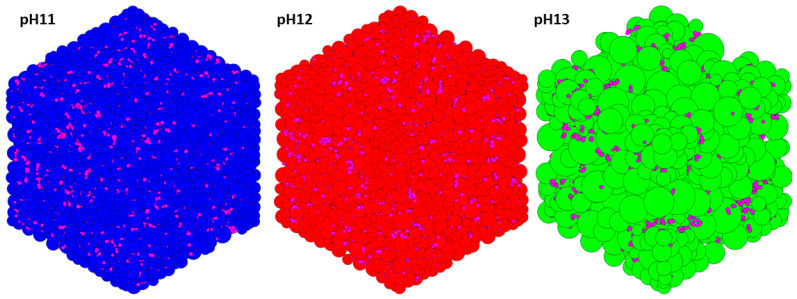
Pore analysis at the end of the simulation. Magenta particles are solids.

**Figure 13:**
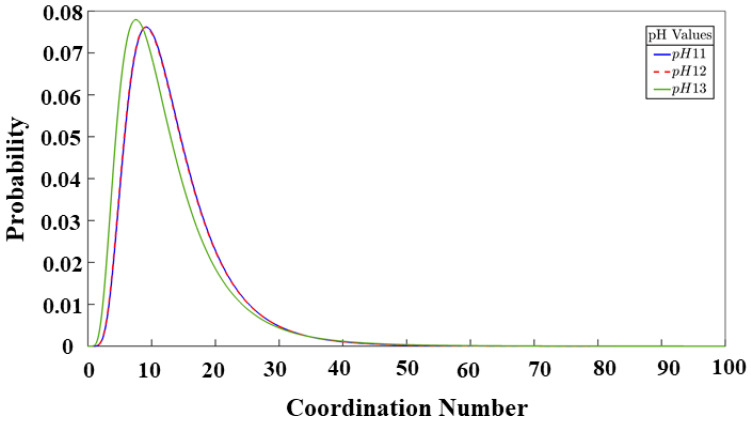
Pore connectivity. In this graphical representation, an analysis of the interconnections among the pores in each system is viable.

Figure 12 depicts the cumulative pore volume observed in each system upon the conclusion of the simulation. The findings showcase a dry density of 2.50 g/cm^3^, 2.46 g/cm^3^, and 2.52 g/cm^3^ for the systems characterized by pH levels 11, 12, and 13, respectively. There is a noticeable trend in which pore dimensions increase with increasing concentrations of the monomers Si(OH)_4_, Al(OH)_4_^−^.Na^+^, and SiO(OH)_3_^−^.Na^+^.3H_2_O, which are crucial for pH stabilization in each system. Figure 13 illustrates the connectivity analysis among pores within each system, revealing a closely aligned trend between the pH 11 and 12 systems. Both demonstrate a prevalent tendency towards pores exhibiting 11 connections. Conversely, the pH 13 system displays a slightly left-shifted curve, indicating a prevalent pore connection trend of 9. This distinction arises from the substantial accumulation of monomers in the system’s final stages, as previously discussed.

**Figure 14:**
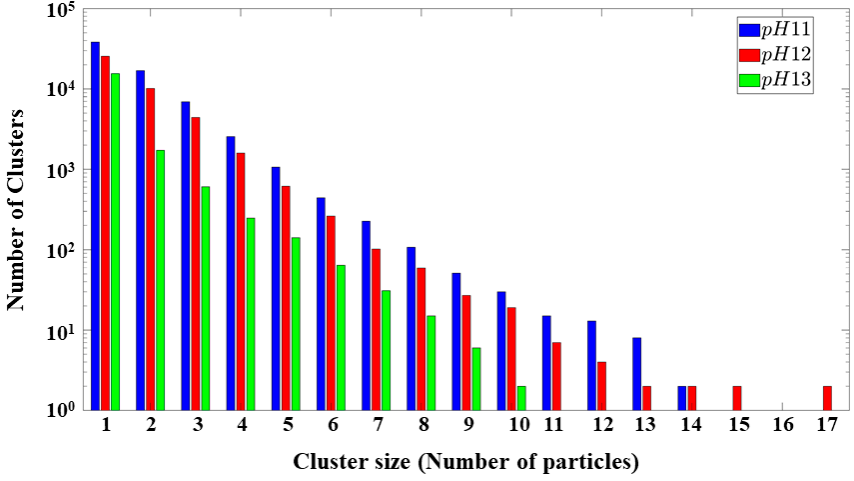
Cluster distribution. The y-axis shows the total number of clusters, and the x-axis describes the number of particles in each cluster. The clusters that have a particle count of 1 are the monomers in the system.

Figure 14 provides a comprehensive analysis of cluster size formation for the three systems. Notably, it reveals a prevailing trend wherein the conclusion of the process demonstrates a higher count of monomers in contrast to the number of clusters. Moreover, a consistent observation across all three systems manifests a predilection for an increased occurrence of clusters composed of two to three particles. These numerical trends elucidate the direct impact of pH variations on the formation and distribution of clusters and pores within the simulated environments.

The future study aims to simulate mesoscale geopolymer composites, incorporating graphene-based nanomaterials [41,42], using the same system as this study did for geopolymer nucleation. It will leverage recent DFT-computed adsorption energies, as reported by Izadifar et al. [43], between graphene-based nanosheets and geopolymers with identical monomer units.

## 4. Conclusions

This research investigated the impact of pH on aluminosilicate gel nucleation using an enhanced off-lattice coarse-grained Monte Carlo (CGMC) computational approach. The tetrahedral geometry of aluminate and silicate monomers, including their binding energy parameters, is obtained from DFT simulations. Key findings include:

1. A methodology for efficient expansion of CGMC system sizes with increased particle counts using the Octree Cell method, reduces waiting times, and harnesses the benefits of parallel high-performance computers.
2. A CGMC methodology to computationally study the critical role of pH in governing dissolution, polycondensation, and reorganization mechanisms in geopolymer materials. As a result, systems with pH levels of 11 and 12 exhibit notably similar behavior regarding the smaller quantity of remaining monomers at the end of simulations, displaying a marginal difference of merely 0.67%. Conversely, the system with pH 13 showcases a higher monomer content by the simulation’s conclusion, depicting a 32.87% increase compared to the pH 11 system and a similar 32.20% increase in contrast to the pH 12 system. The same values are represented for the behavior of the monomers in the polycondensation process; nevertheless, a difference of 2,697,600 (out of total 56 millions iterations) iterations is identified between the systems with pH 11 and 12 to reach the equilibrium between particles contributing to cluster formation and those persisting as monomers throughout the simulation. It is evident that the pH 11 system, whith the largest number of particles among pH 11 to 13), achieved equilibrium in the shortest duration.
3. Digital image analysis of porosity revealed a direct link between higher pH levels, increased porosity, and the prevalence of monomer species. Similarities in the polycondensation processes of systems at pH 11 and 12 tend to coarsen the final pore structure with increasing pH. The comparative analysis between systems 11 and 12 shows an observed increase of 10.31% in pore dimensions, while the comparison between systems 11 and 13 shows a discernible increase of 32.53% in pore dimensions. pH 13 contains pores with larger diameters, in agreement with the higher percentage of monomers at the end of the simulation.

## Figures and Tables

**Table 1:** Composition of the geopolymer-forming system based on the results reported by White et al. and the silicate thermodynamic speciation model [10].

	Percentage (%)		
pH System	Water + Na	Silicate in Solution	Metakaolinite
11 12 13	68.4%	10.6	21%

**Table 2:** The total number of silicate particles and metakaolinite particles presented for three different pH (11, 12, and 13) systems at the beginning of the simulation.

Number of Particles		
pH	Silicate in Solution	Metakaolinite
11	17,560	97,336
12	10,640	64,000
13	5824	17,576

**Table 3:** The total number of each silicate particle present in three different pH systems at the beginning of the simulation.

	pH Systems		
Silicate Monomer Species	11	12	13
**M**	872	0	0
**M^−^.Na^+^.3H_2_O**	15,816	8512	1744
**M^2−^.2Na^+^.6H_2_O**	872	2128	4080

M = Si(OH)_4_, M^−^ = SiO(OH)_3_^−^, M^2−^ = SiO_2_(OH)_2_^2−^.

## Data Availability

The data presented in this study are available on request from the corresponding authors.

## Appendix A

**Table A1:** Bond lengths defining the coarse-grained particle size for each particle, resulting in the dimerization reactions of Si-O-Al and Si-O-Si, as denoted in Figure 2.

Atoms	Bond Length (Angstrom)
**O1-Si1**	1.64
**O2-Si1**	1.64
**O3-Si1**	1.66
**O4-Si1**	1.63
**O4-Si2**	1.64
**O5-Si2**	1.64
**O6-Si2**	1.64
**O7-Si2**	1.64
**O8-Al1**	1.76
**O9-Al1**	1.76
**O10-Al1**	1.77
**O11-Al1**	1.76
**O11-Si3**	1.63
**O12-Si3**	1.65
**O13-Si3**	1.65
**O14-Si3**	1.64

**Table A2:** Angles between atoms defining the coarse-grained particle size for each particle, resulting in the dimerization reaction of Si-O-Al and Si-O-Si, as denoted in Figure 2.

Atoms	Angle (Degree)
**O1-Si1-O2**	106.74°
**O2-Si1-O3**	106.97°
**O3-Si1-O4**	104.25°
**O4-Si1-O1**	110.56°
**Si1-O4-Si2**	138.28°
**O4-Si2-O5**	104.21°
**O5-Si2-O6**	107.15°
**O6-Si2-O7**	108.41°
**O7-Si2-O4**	109.46°
**O8-Al1-O9**	107.72°
**O9-Al1-O10**	110.84°
**O10-Al1-O11**	102.20°
**O11-Al1-O8**	111.33°
**Al1-O11-Si3**	135.12°
**O11-Si3-O12**	109.02°
**O12-Si3-O13**	104.84°
**O13-Si3-O14**	111.35°
**O14-Si3-O11**	110.30°
